# Candidate Oligo Therapeutic Target, miR-330-3p, Induces Tamoxifen Resistance in Estrogen Receptor-Positive Breast Cancer Cells via HDAC4

**DOI:** 10.1155/2023/2875972

**Published:** 2023-09-06

**Authors:** Meng Zhang, Mei Wang, Zhiming Jiang, Ziyi Fu, Jingjing Ma, Sheng Gao

**Affiliations:** ^1^Division of Breast Surgery, Department of General Surgery, Nanjing Drum Tower Hospital, The Affiliated Hospital of Nanjing University Medical School, Nanjing 210008, China; ^2^Department of Pathology, Nanjing Hospital of Chinese Medicine Affiliated to Nanjing University of Chinese Medicine, Nanjing 210001, China; ^3^Department of Ultrasound Medicine, Nanjing Drum Tower Hospital, The Affiliated Hospital of Nanjing University Medical School, Nanjing 210008, China; ^4^Department of Breast Disease Research Center, The First Affiliated Hospital, Nanjing Medical University, Nanjing 210029, China; ^5^Nanjing Maternal and Child Health Institute, Nanjing Maternal and Child Health Care Hospital, Obstetrics and Gynecology Hospital Affiliated to Nanjing Medical University, Nanjing 210004, China

## Abstract

Tamoxifen is a drug used for treating breast cancer (BC), especially for individuals diagnosed with estrogen receptor-positive (ER+) BC. Its prolonged use could reduce the risk of recurrence and significantly lengthen the survival rate of BC patients. However, an increasing number of patients developed resistance to tamoxifen treatment, which reduced therapeutic efficiency and caused substandard prognosis. Therefore, the exploration of the molecular processes involved in tamoxifen resistance (TR) is urgently required. This investigation aimed to elucidate the relationship of microRNA-330 (miR-330-3p) with the TR of BC. There is little information on miR-330-3p′s link with drug-resistant BC, although it is well known to regulate cell proliferation and apoptosis. Primarily, miR-330-3p expression in parental BC (MCF7/T47D), TR (MCF7-TR), and T47D/TR cell lines was detected by qRT-PCR. Then, the impact of miR-330-3p on the TR of BC cells was assessed by a cell proliferation assay. Lastly, dual-luciferase reporter, qRT-PCR, and western blot assessments were carried out to identify histone deacetylase 4 (HDAC4) as the potential miR-330-3p target gene. The data indicated that miRNA-330 was overexpressed in TR ER+ BC cells and its overexpression could induce TR. Furthermore, miRNA-330 could also reduce the expression of HDAC4, which is closely linked to TR, and overexpression of HDAC4 could reverse miRNA-330-induced drug resistance. In summary, miR-330-3p could induce TR of ER+ BC cells by downregulating HDAC4 expression, which might be a novel marker of TR and a possible treatment target against BC patients who are tamoxifen-resistant.

## 1. Introduction

In women, breast cancer (BC) is the most frequent and threatening cancer type, accounting for 7–10% of all malignant tumors. BC incidence has increased since the late 1970s [1, 2]. Estrogen receptor positive (ER+) has been reported to be expressed in 70–80% of BC patients, with increasing incidence every year [3, 4]. BC has substantially endangered women's mental and physical health; therefore, the investigation of BC has attracted the attention of scientists worldwide.

Tamoxifen is a nonsteroidal antihormonal antineoplastic agent [5–7], is used for the clinical treatment of BC, and is particularly effective in ER+ patients. Tamoxifen could significantly alleviate the recurrence risk of BC and is very effective among metastatic ER+ BC patients [8, 9]. Long-term use of tamoxifen could prolong survival and reduce recurrence in BC patients. However, more and more patients gradually developed tamoxifen resistance (TR) in recent years [10]. TR has been reported in 30 to 40% of BC patients treated for 5 years. Cancer cells are generally more aggressive when they get resistant and more difficult to treat with conventional therapies, making it even worse to continue treatment [11, 12]. Therefore, TR is very important for treating BC. Understanding the bio-molecular mechanism of tamoxifen can solve the drug-resistance problem.

MicroRNA (miRNA) is an evolutionarily conservative noncoding small RNA comprising approximately 20 nucleotides [13–15]. Recently, it has been suggested that these miRNAs correlate with the initiation and progression of multiple tumors, including breast, lung, and gastric cancers [16–21]. It has been demonstrated that miRNAs might be essential bio-indices and treatment targets for clinical detection and cancer therapy. Moreover, several reports have revealed a close relationship between miRNAs in TR [22]. miRNA-181b and miRNA301a were found to be aberrantly expressed in tamoxifen-resistant BC. Furthermore, overexpression of miRNA375, miRNA221/222, miRNA101, miRNA519a, miRNA451, miRNA575, miRNA32-5p, miRNA195, and miRNA497 could induce TR [23–29]. Therefore, it is necessary to deeply understand miRNAs' potential activity and function in TR.

This investigation focuses on elucidating the link between miR-330-3p and TR. It was revealed that miR-330-3p upregulation was substantially related to TR and that miR-330-3p could cause drug resistance by downregulating HDAC4. This investigation might provide useful information to find new therapeutic targets and strategies for TR BC patients.

## 2. Materials and Methods

### 2.1. Reagents and Cell Lineages

The human BC cell lineages (T47D and MCF7) were provided by the American Type Culture Collection (ATCC, USA) and propagated in DMEM (Gibco, Invitrogen China Limited, China) and RPMI-1640 Wisent (China), respectively, augmented with 10% fetal bovine serum (FBS, Gibco), 1 mM sodium pyruvate, and 100 U/mL penicillin and streptomycin (Gibco), and incubated at 37°C in 5% CO_2_. The medium was replaced every 48 hours.

Antibodies used were *β*-actin (Sigma-Aldrich), HDAC4 (#15164, 140kd, Cell Signaling Technology, USA), and Lipo2000 (Invitrogen, USA).

### 2.2. Cell Counting Kit-8 (CCK-8) Assay

Cell viability was elucidated by CCK-8 (Dojindo Laboratories, Kumamoto, Japan). Cells were propagated for 24h, the media were refreshed by only RPMI-1640, and then 150 nM miR-330-3p mimics and miR-330-3p sponge (negative control; NC) were transfected via the Lipo 2000. After 48 h, 3000 cells/wells were propagated in 96-well plates in 100 *μ*L of media +10% FBS at 37°C for 24 h. Then, the supernatant was refreshed by 200 *μ*L full growth medium with tamoxifen; each treatment had three replicates. Then, 10 *μ*L of CCK-8 solution was incorporated in the medium for 2 h at 37°C, and their absorbance (OD) was measured via a microplate spectrophotometer (Biotek, Germany) at 490 nm. Each sample's OD was subtracted from the blank value, and cell growth reduction was measured as a percentage of control OD (no drug). The data are depicted as the mean ± SD for repeated experiments thrice.

### 2.3. Dual-Luciferase Reporter Assays

Cells at the concentration of 10000/well were propagated in 48-well plates overnight in 100 *μ*L of media +10% FBS. Then, cells were co-transfected by HDAC4 3′-UTR luciferase vector and pre-miR-330-3p or miRNA negative control via DharmaFECT Duo Transfection Reagent (Dharmacon) for 48h at 37°C and 5% CO_2_. Then, with the help of phosphate-buffered saline (PBS), cells were rinsed before lysis with 100 ul PLB lysis solution and collection cracking fluid. The luciferase function was tested via the Dual-Luciferase Reporter Assay System (Promega, USA) and a luminometer. The experiments were repeated thrice.

### 2.4. RNA Extraction and Real-Time Quantitative PCR

For obtaining whole RNA, Trizol (Takara, Japan) reagent was utilized by following the instructions provided by the manufacturer (Invitrogen, CA, USA) [30]. Quantity and quality were assessed via Nano-drop and Agilent 2100 Bio-analyzer (Agilent Technologies). Then, purified RNA (1 *μ*g) was reversely transcribed with the help of PrimeScript RT Reagent Kit (Takara, Japan). cDNA and total RNA were quantified via a biophotometer (Eppendorf, Germany). The acquired cDNA was stacked at −20°C. The 20 *μ*L reactions were prepared in 96-well plates comprising 10 *μ*L of SYBR Green PCR Master Mix (Takara, Japan), 1 *μ*L of each primer (2 *μ*M) (supporting information (SI) Table S1), and 8 *μ*L of template DNA. *β*-actin was utilized as an endogenous gene. Each reaction was repeated thrice. The relative expression levels were elucidated by the 2^−^^^^Ct^ method.

### 2.5. Western Blot Assessment

Proteins were acquired by lysing cells with RIPA buffer. Then, standard Western blot protocol was conducted to elucidate protein expression. Briefly, with the help of KEYGEN Protein Extraction Kit (KEYGEN, China), protein samples were obtained at the indicated time point, boiled in sample buffer comprising sodium dodecyl sulfate (5 *∗* SDS) for 5 minutes, exposed to 8% SDS-PAGE electrophoresis, and then translocated to PVDF (polyvinylidene fluoride) membranes (Bio-Rad), which were then blocked for 2 hours in 5% milk-Tris-Buffered Saline Tween-20 (TBST) at ambient temperature, kept with HDAC4 and *β*-actin (Cell Signaling Technology, USA) monoclonal antibodies at 4°C overnight, washed thrice in TBST, and incubated again for 2 hours in appropriate horseradish peroxidase-linked secondary antibodies (Cell Signaling Technology, USA) at ambient temperature. Enhanced chemiluminescence (Thermo, USA) was utilized for visualizing blots and assessed via the scanning densitometer using molecular analysis software FluorChem M system (Protein Simple, USA).

### 2.6. Statistics

The variabilities in protein levels and invasion rate between the treated and control cells were elucidated by one-way ANOVA and standard student *t*-test. SPSS (20 version) (SPSS Inc. Chicago, Illinois, USA) was utilized for statistical measurements. *P* value <0.05 was termed statistically important. Each experiment was repeated thrice.

## 3. Results

### 3.1. ER+ Breast Cancer Cell's Sensitivity to Tamoxifen

**Figure 1:**
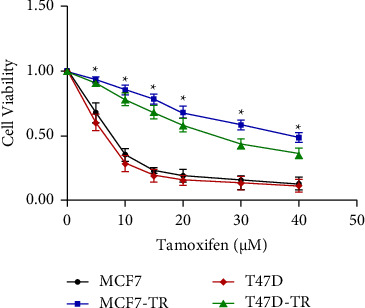
Tamoxifen-resistant cells were constructed based on parental cells with tamoxifen treatment. CCK-8 test was performed to identify the vitality of acquired tamoxifen-resistant breast cancer cell lines (MCF7/TR and T47D/TR) and their parental cell lines (MCF7 and T47D). Cells were treated with the indicated tamoxifen dose for 72 h (data are represented as mean ± S.D of triplicate experiments; *p* < 0.05).

To elucidate the undergoing TR mechanism in ER+ BC cells, resistant BC cells (T47D-TR and MCF7-TR) against tamoxifen were constructed. The drug susceptibility of MCF7, T47D, T47D-TR, and MCF7-TR cells was assessed by elucidating their viability after the tamoxifen dose. The cell proliferation assay results showed that the survival ratio of T47D-TR and MCF7-TR cells is much higher than that of parental cell lines. The effective concentrations (EC50) were calculated separately for MCF7 (EC50 = 7.24 *μ*M), MCF7-TR (EC50 = 39.84 *μ*M), T47D (EC50 = 6.86 *μ*M), and T47D-TR (EC50 = 28.12 *μ*M) cells, indicating the construction of enhanced resistant cell lines (Figure 1).

### 3.2. miR-330-3p Is Highly Expressed in Tamoxifen-Resistant Cell Lines

**Figure 2:**
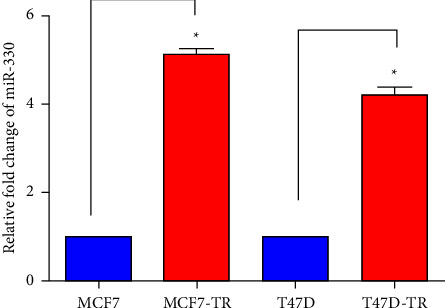
Endogenous expression levels of miR-330-3p in breast cancer cells. qPCR results showed that miR-330-3p is highly expressed in tamoxifen-resistant MCF7-TR and T47D-TR cells (data are represented as mean ± S.D of triplicate experiments; *p* < 0.05).

Research suggests that miR-330-3p is closely linked with cell proliferation and apoptosis [31–33]. To investigate whether its expression is associated with the sensitivity of ER+ BC cells to tamoxifen, real-time RT-PCR identified miR-330-3p in constructed tamoxifen-resistant and parental cell lines. The data revealed that its levels in T47D-TR and MCF7-TR cells were notably increased than in T47D and MCF7 cells. Its expression in MCF7-TR cells was 5.12 times more than that in MCF7 cells, and in T47D-TR cells, it was about 4.21 times increased than that in T47D cells (Figure 2). Furthermore, miR-330-3p was upregulated in tamoxifen-resistant cells, expressing a positive correlation with the ER+ BC cells' TR.

### 3.3. miR-330-3p Induced Tamoxifen Resistance in Breast Cancer

**Figure 3:**
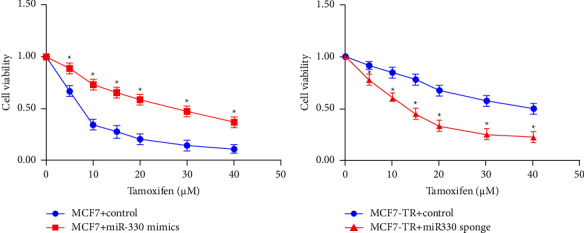
miR-330-3p regulates tamoxifen resistance in breast cancer. (a) Overexpression of miR-330-3p increased tamoxifen resistance in the parental cell lines. (b) miR-330-3p inhibition decreased the resistance of MCF7-TR cells to tamoxifen (data are represented as mean ± S.D of triplicate experiments; *p* < 0.05).

To confirm the brief impact of miR-330-3p on tamoxifen sensitivity of BC cells, miR-330-3p mimics (Ribobio, China) were transfected in MCF7 cells, which revealed that miR-330-3p upregulation enhanced cell viability after tamoxifen treatment (Figure 3(a)), indicating that miR-330-3p upregulation alleviated the sensitivity of MCF7 cells to tamoxifen. miR-330-3p sponge inhibitor (Ribobio, China) transfected MCF7-TR revealed depression of miR-330-3p, which decreased cell viability with tamoxifen therapy (Figure 3(b)). EC50 of cells was upregulated from 7.02 *μ*M (MCF7+ control) to 29.48 *μ*M (MCF7+ miR-330 mimics), meanwhile downregulated from 40.26 *μ*M (MCF7-TR+ control) to 14.51 *μ*M (MCF7-TR+ miR-330 sponge), indicating that miR-330-3p downregulation increases MCF7-TR cells sensitivity to tamoxifen.

### 3.4. Gene Ontology and Pathway Analysis of miR-330-3p Target Genes

**Figure 4:**
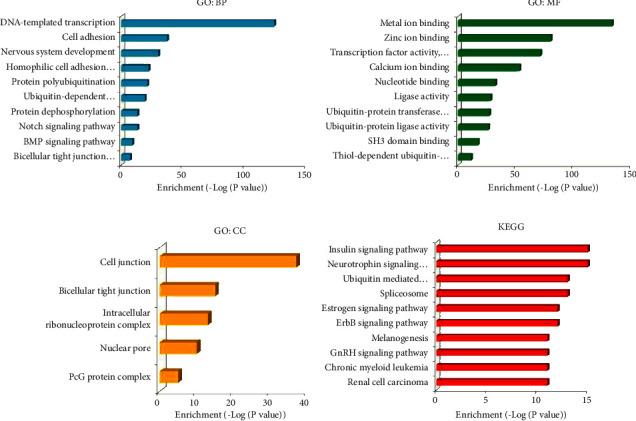
Gene ontology and pathway analysis of miR-330-3p. The potential mechanism involved in miR-330-3p induced tamoxifen resistance as predicted after analyzing the roles of miR-330-3p targeted substrates. (a) miR-330-3p participates in DNA-template transcription, cell adhesion, and nervous system development biological processes. (b) miR-330-3p possesses the ability to regulate metal ion binding, zinc ion binding, and transcription factor activity. (c) The downstream products are mainly located on cell junction, bicellular tight junction, intracellular ribonucleoprotein complex, nuclear pore, and PcG protein complex. (d) The targeted genes were enriched in insulin/neurotrophin/estrogen/ErbB/GnRH signaling pathways and ubiquitin-mediated proteolysis.

The target prediction of miR-330-3p was performed, and bioinformatics tools assessed its function. Analyzed GO data suggested that targeted genes were enriched in the biological processes of DNA-templated transcription, nervous system development, cell adhesion, ubiquitin-dependent protein catabolic process, Notch signaling pathway, protein dephosphorylation, protein poly-ubiquitination, homophilic cell adhesion via plasma membrane adhesion molecules, BMP signaling pathway, and bicellular tight junction assembly (Figure 4(a)). The target genes' molecular functions included zinc, calcium, and metal ion binding, ubiquitin-protein ligase activity, transcription factor activity, nucleotide binding, ubiquitin-protein transferase activity, ligase activity, sequence-specific DNA binding, thiol-dependent ubiquitin-specific protease activity, and SH3 domain binding, (Figure 4(b)). The product of these genes is primarily composed of cell junction, bicellular tight junction, intracellular ribonucleoprotein complex, nuclear pore, and PcG protein complex (Figure 4(c)). These genes were classified into different biological pathways, including signaling pathways of insulin, neurotrophin, estrogen, ErbB, and GnRH and pathways of chronic myeloid leukemia, ubiquitin mediated proteolysis, spliceosome, melanogenesis, and renal cell carcinoma (Figure 4(d)).

### 3.5. miR-330-3p Reduced HDAC4 Expression by Targeting the 3′-UTR Region of HDAC4 mRNA

**Figure 5:**
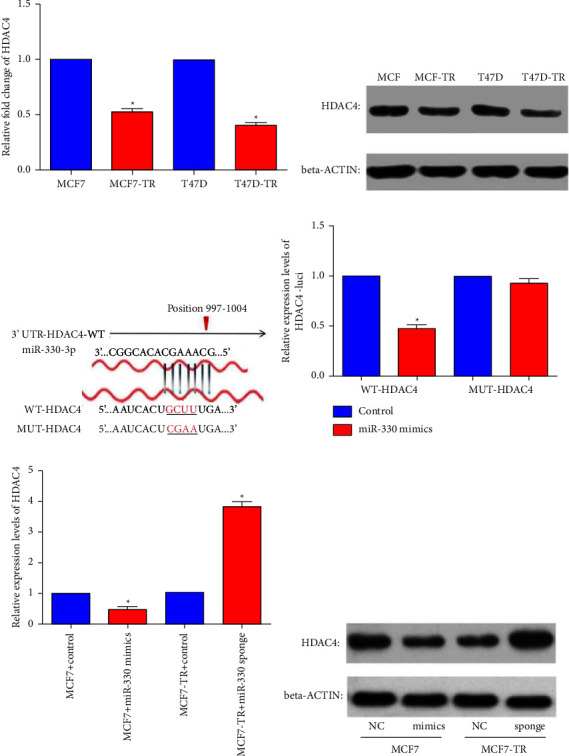
miR-330-3p reduced the expression of HDAC4 by targeting the 3′-UTR region of HDAC4 mRNA. (a, b) Both RNA and protein expression levels were dysregulated in tamoxifen-resistant breast cancer cell lines. (c) Potential binding sites of miR-330-3p on HDAC4-mRNA. (d) Dual-luciferase reporter assay confirmed the binding sites of miR-330-3p on HDAC4-mRNA. (e) miR-330-3p could regulate HDAC4 mRNA expression directly. (f) Inconsistency with RNA level and miR-330-3p regulated HDAC4 protein expression (data are represented as mean ± S.D of triplicate experiments; *p* < 0.05).

HDAC4 could interact with ER*α* N terminus in the nucleus and then suppress transcriptional activity of estrogen-responsive genes by estrogen and selective ER modulators, including tamoxifen [34, 35]. This investigation predicted HDAC4 as an efficient miR-330-3p target and confirmed that both HDAC4 mRNA (Figure 5(a)) and protein (Figure 5(b)) levels alleviate in tamoxifen-resistant cell lines. It was also predicted that miR-330-3p might attach to the “UGCUUUG” of HDAC4-mRNA 3′-UTR region through the “ACGAAAC” core sequence. To confirm miR-330-3p binding regions within the 3′-UTR site of HDAC4-mRNA, a wild genotype (wt)/mutant genotype (mut) of the miR-330-3p-binding site in HDAC4 3′-UTR was constructed (Figure 5(c)). Then, the expression levels of wt and mut constructs were evaluated based on miR-330-3p overexpression in MCF7 cells (mimics group) via the dual-luciferase analysis system (Figure 5(d)). Consistently, miR-330-3p upregulation could alleviate both the protein and mRNA levels of HDAC4, and its inhibition enhances HDAC4 expression (Figures 5(e) and 5(f)). Altogether, the data indicate that miR-330-3p silences HDAC4 expression by targeting the 3′-UTR site of HDAC4 mRNA in ER+ BC cells.

### 3.6. miR-330-3p Induces Tamoxifen Resistance in ER+ Breast Cancer through HDAC4 Downregulation

**Figure 6:**
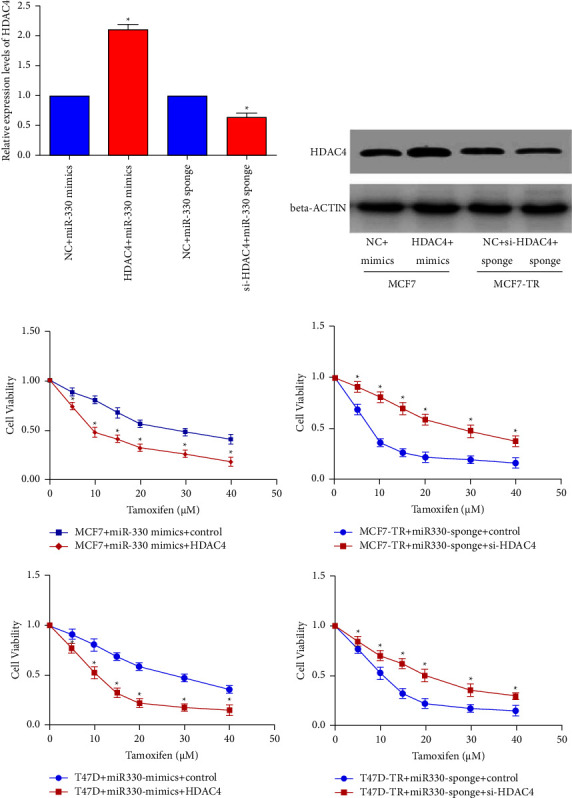
miR-330-3p induces tamoxifen resistance in ER+ breast cancer through downregulating HDAC4. (a, b) MCF7 cells were co-transfected with miR-330-3p mimics and HDAC4 vector or scramble control (NC). MCF7-TR cells were co-transfected with miR-330-3p sponge inhibitor and HDAC4-siRNA (si-HDAC4) or scrambled control (NC). (a) qRT-PCR was performed to detect the mRNA expression of HDAC4. (b) Western blotting identified the protein expression of HDAC4. (c) Co-transfection of miR-330-3p and HDAC4 was carried out in parental cell lines MCF7 and T47D; co-transfection of miR-330-3p sponge inhibitor and HDAC4-siRNA was carried out in resistant MCF7-TR and T47D-TR cell lines. Subsequently, cells were treated with the indicated dose of tamoxifen for 72 h and CCK-8 assay was performed to test cell viability. miR-330-3p could induce tamoxifen resistance in ER+ breast cancer cells through HDAC4 (data are represented as mean ± S.D of triplicate experiments; *p* < 0.05).

The rescue strategy elucidated HDAC4 activity in the process of miR-330-3p-induced TR. MCF7 cells were co-transfected with miR-330-3p mimics/HDAC4 vector or mimics/empty vector (pcDNA-3.1-HDAC4 or pcDNA-3.1). In contrast, MCF7-TR cells were co-transfected with miR-330-3p sponge/HDAC4-siRNA or sponge/scramble control, respectively. The HDAC4 levels were assessed by qRT-PCR (Figure 6(a)) and western blot techniques (Figure 6(b)). It was indicated that miR-330-3p-induced TR was reversed after HDAC4 overexpression in parental cells; in contrast, HDAC4 silencing dramatically restored TR after blocking miR-330-3p in resistant cell lines (Figure 6(c)). Compared to the control group, HDAC4 overexpression decreased EC50 of MCF7 cells from 30.01 *μ*M (MCF7+ miR-330 mimics+ control) to 9.79 *μ*M (MCF7+ miR-330 mimics+ HDAC4), while HDAC4 suppression increased EC50 of MCF7 cells from 8.14 *μ*M (MCF7-TR+ miR-330 sponge+ control) to 28.23 *μ*M (MCF7-TR+ miR-330 sponge+ si-HDAC4). As the same, HDAC4 overexpression decreased EC50 of T47D cells from 29.16 *μ*M (T47D+ miR-330 mimics+ control) to 10.05 *μ*M (T47D+ miR-330 mimics+ HDAC4), while HDAC4 suppression increased EC50 of T47D cells from 10.21 *μ*M (T47D-TR+ miR-330 sponge+ control) to 19.88 *μ*M (T47D-TR+ miR-330 sponge+ si-HDAC4). These results demonstrated that miR-330-3p could induce TR through downregulation of HDAC4 expression in ER+ BC cells.

## 4. Discussion

Breast cancer ranks 1^st^ among the most malignant tumors threatening women's health. Recently, it has been identified that the incidence of BC is gradually increasing, especially in ER+ patients. Tamoxifen is the first-line agent against ER+ BC [36, 37]. However, the recurrence increases after 10 years of tamoxifen treatment without clear rationales [38, 39]. The growing number of drug-resistant patients causes clinical therapeutic obstacles and greatly burdens society. Clinical trials have revealed that most patients could benefit from novel drugs which uncover drug resistance. FDA has approved 10 oligo agents for clinical use. This investigation aimed to elucidate the possible roles of highly expressed miR-330-3p in tamoxifen-resistant BC (Figure 2). The Tam-resistant cells used here showed less sensitivity to tamoxifen, suggesting that it is better to use 4OH-Tam, a more active derivative [40, 41].

The literature suggests that based on the cell types, miRNAs play multi-roles in regulating cellular processes. The literature reveals that miR-330-3p induces tumor progression in various cancers, including nonsmall-cell lung cancer, gastric cancer, colorectal cancer, and pancreatic cancer. It also promotes BC cell migration through CCBE1, Myc, and PDCD4 pathways, predicting substandard prognosis in BC patients. This investigation reveals that miR-330-3p overexpression could induce TR in ER+ BC cells (Figure 3); however, the underlying information on the mechanism is still limited.

Based on the rationales of miRNAs negatively regulating target genes, possible miR-330-3p targets were predicted via target scan e-tools, and then gene ontology and pathway analysis were carried out in the David database. The data suggested that miR-330-3p might regulate diverse biological processes through neurotrophin signaling, spliceosome, insulin signaling, estrogen signaling, ubiquitin-mediated proteolysis, and ErbB signaling pathways (Figure 4). Furthermore, HDAC4 was determined as the miR-330-3p target gene and was involved in miR-330-3p-mediated TR of ER+ BC cells (Figures 5(a) and 5(b)). It is essential to elucidate how miR-330-3p regulates HDAC4 expression and induces TR to better understand and explore novel strategies to solve this clinical obstacle.

HDAC4, a key member of the classic HDAC family, regulates transcriptional activity by modulating histone in the nucleus [42]. Aberrant HDAC4 expression has been correlated with multiple biological processes in cancers, including tumorigenesis, migration, and drug resistance. Recently, HDAC4 has been found to increase gastric cisplatin resistance via the p53-p73/BIK pathway [43]. In addition, HDAC4 might cause 5-FU resistance in BC cells through deacetylation of the SMAD4 promoter [44]. Also, HDAC4 interacts with ER*α* N-terminus in the nucleus and then suppresses the transcription activity of estrogen-responsive genes by estrogen and tamoxifen [34]. Moreover, Ahmad et al. found that miR-10b could induce HDAC4-mRNA degradation, resulting in BC cell's TR [35].

Multiple binding sites exist in the same gene's 3′-UTR for different miRNAs. This investigation identified that despite miR-10b, miR-330-3p modified HDAC4 expression by targeting the 3′-UTR region of HDAC4 mRNA in ER+ BC cells (Figure 5). To further ensure HDAC4 function in miR-330-3p-induced TR, its impact on TR was assessed based on HDAC4 knock-down in ER+ BC cells. The data confirmed that miR-330-3p increases ER+ BC cells' TR by reducing HDAC4 (Figure 6).

## 5. Conclusion

In conclusion, it was revealed that aberrant miR-330-3p expression could increase ER+ BC resistance to tamoxifen, that miR-330-3p levels reversely correlate with HDAC4 in ER+ BC cells, and that miR-330-3p induces TR in an HDAC4-dependent manner. This research provides brief information that explores the underlying mechanism of BC cells' TR and indicates that miR-330-3p might be a prognostic index for ER+ BC patients and could be a candidate therapeutic target to overcome TR.

## Data Availability

The data generated or analyzed during this study are included within the article.

## Supplementary Materials

Supplementary Materials Table S1: Primer sequences used for qRT-PCR.
