# Supplementary material for: Candidate Oligo Therapeutic Target, miR-330-3p, Induces Tamoxifen Resistance in Estrogen Receptor-Positive Breast Cancer Cells via HDAC4
Source: Breast J. 2023 Sep 6;2023:2875972. doi: 10.1155/2023/2875972 (PMC10499526; doi:10.1155/2023/2875972)
Supplement: Supplementary Materials — Table S1: Primer sequences used for qRT-PCR. [file 2875972.f1.docx]

**Table S1. Primers for qRT-PCR**

| Gene | Forward (5’-3’) | Reverse (5’-3’) |
| --- | --- | --- |
| miR-330-3p | TGCGGTCTCTGCAGGCCGTGTGCT | CCAGTGCAGGGTCCGAGGT |
| U6 | TGCGGGTGCTCGCTTCGGCAGC | CCAGTGCAGGGTCCGAGGT |
| HDAC4 | CACACACTCCTCTACGGCACAAATC | ACCTTGAAGACCAGCTCCACTACACA |
| β-actin | CCTCGCCTTTGCCGATCC | GGATCTTCATGAGGTAGTCAGTC |
